# The Bimanual Observation of The Hands (BOTH): Development, reliability, and validity for stroke rehabilitation

**DOI:** 10.1371/journal.pone.0316356

**Published:** 2025-01-07

**Authors:** Debbie Rand, Nitsan Omer, Amihai Levkoviz, Samar Assadi Khalil

**Affiliations:** 1 Department of Occupational Therapy, School of Health Professions, Faculty of Medical and Health Sciences, Tel Aviv University, Tel Aviv, Israel; 2 Benai Zion Medical Center, Haifa, Israel; Shahid Chamran University of Ahvaz, ISLAMIC REPUBLIC OF IRAN

## Abstract

**Importance:**

To efficiently perform bimanual daily tasks, bimanual coordination is needed. Bimanual coordination is the interaction between an individual’s hands, which may be impaired post-stroke, however clinical and functional assessments are lacking and research is limited.

**Objectives:**

To develop a valid and reliable observation tool to assess bimanual coordination of individuals post-stroke.

**Design:**

A cross-sectional study.

**Setting:**

Rehabilitation settings.

**Participants:**

Occupational therapists (OTs) with stroke rehabilitation experience and individuals post stroke.

**Outcomes and measures:**

The development and content validity of BOTH included a literature review, review of existing tools and followed a 10-step process. The conceptual and operational definitions of bimanual coordination were defined as well as scoring criteria. Then multiple rounds of feedback from expert OTs were performed. OTs reviewed BOTH using the ‘Template for assessing content validity through expert judgement’ questionnaire. Then, BOTH was administered to 51 participants post-stroke. Cronbach’s alpha was used to verify internal reliability of BOTH and construct validity of BOTH was assessed by correlating it to the bimanual subtests of The Purdue Pegboard Test.

**Results:**

Expert validity was established in two-rounds with 11 OTs. Cronbach’s alpha was α = 0.923 for the asymmetrical items, 0.897 for the symmetrical items and 0.949 for all eight items. The item-total correlations of BOTH were also strong and significant. The total score of BOTH was strongly significantly correlated with The Purdue–Both hands placement (r = .787, p < .001) and Assembly (r = .730, p < .001) subtests.

**Conclusions and relevance:**

BOTH is a new observation tool to assess bimanual coordination post-stroke. Expert validity of BOTH was established, excellent internal reliability and construct validity were demonstrated. Further research is needed, so in the future, BOTH can be used for clinical and research purposes to address bimanual coordination post-stroke.

## Introduction

Most daily activities involve bimanual tasks that require the use of both hands, such as peeling a cucumber, tying shoelaces, and wrapping a gift [1]. The performance of bimanual activity may be impaired post-stroke due to motor impairments and functional difficulties of the affected upper extremity. Functional ability of the stroke-affected upper extremity, which is often assessed utilizing bimanual tasks, is correlated to impairments of this upper extremity [2–8]. The ABILHAND [2], for example, is a self-report questionnaire used to measure the perceived difficulty to perform a wide range of daily bimanual activities (e.g., threading a needle, fastening a zipper, and unwrapping a chocolate bar). Using the ABILHAND, activities that require fine manual dexterity were rated as ‘difficult’ or ‘impossible to perform’ by 75 individuals with stroke [3]^.^ The Adult Assisting Hand Assessment Stroke (Ad-AHA-Stroke) [4] is an observation-based bimanual upper extremity performance measurement tool to assess spontaneous use of the affected hand during the execution of a bimanual task (gift wrapping or making a sandwich). Poor bimanual activity of 89 participants 3-weeks post-stroke, improved over time and was correlated with the motor impairment at each time point of a longitudinal study [5]. The Chedoke Arm and Hand Activity Inventory (CAHAI) [6] requires performing bimanual activities, such as opening a jar and applying toothpaste to a toothbrush, but it also assesses the functional ability only of the stroke affected upper extremity. The role of the affected upper extremity (i.e. holds jar or holds the lid) and the amount of assistance that this hand requires are also recorded. The Yonsei-Bilateral Activity Test (Y-BAT) [7] is a 19-item observation tool to assess the quality of bimanual upper extremity function. Its use is recommended before and after bilateral upper extremity intervention to improve function of the affected upper extremity. The Bimanual Assessment Measure (BAM) [8] was recently developed for individuals with chronic stroke to assess their ability to perform 11 bimanual tasks such as zipping up a zipper and holding a tray. By observing the participant performing the tasks, the role (stabilizer or manipulator) of the affected upper extremity is identified and the performance (spontaneously, timing, skill) of this upper extremity is rated. While assessing motor and functional abilities of the affected upper extremity is essential, the assessment of bimanual coordination is also important.

Bimanual coordination is essential for efficiently performing daily tasks. It is the coordination and interaction between an individual’s hands needed to efficiently perform daily bimanual tasks. Different aspects of bimanual coordination, such the timing of hand movements, the location in space, and the generation of force, have been found to be impaired post-stroke [9–11]. A few recent studies have addressed bimanual coordination post-stroke in the laboratory or by using sensors, but these are not accessible to clinicians. Duff et al.(2022) [12] developed a sensitive method to identify upper extremity intra and inter-limb coordination during task performance. They calculated a paretic/non-paretic arm use ratio from wrist sensors for various kinematic measures (acceleration, angular rate of change, orientation) while performing unimanual, bimanual symmetrical, and bimanual asymmetrical tasks. Correlations were found between coordination (sensor data) to spontaneous hand-use (assessed by the Ad-AHA Stroke) and motor ability of individuals with stroke and healthy controls. Differences in upper extremity use for different types of tasks were found within and between groups and this was correlated with the clinical scores. In another study, the coordination between alternately performing pronation-supination of both forearms was assessed using kinematic measurements of coordination using the Interlimb Coordination Test from the Comprehensive Coordination Scale [11]. The Comprehensive Coordination Scale [13] is a newly developed observation to assess motor coordination of multiple body segments for adults with neurological conditions. Impaired coordination was found in 13 individuals with stroke compared with 13 healthy controls using this new tool. Another study found that individuals with stroke have impaired ability to perform a bimanual task (the Purdue Assembly task) and diminished bimanual force coordination (e.g., while tracking a trapezoid trajectory) compared to healthy controls [10]. In another study, the kinematics of the bimanual coordination of 12 individuals with stroke was found to improve over time during a bimanual and unilateral reach-to-grasp task [14].

These small studies allow some understanding regarding impaired bimanual coordination post-stroke, but it is still unclear how impaired bimanual coordination impacts daily performance [9]. Bimanual coordination has even been termed ‘the missing piece on arm rehabilitation post-stroke’ [15], because it is not usually researched. Most studies post-stroke focus either on the affected upper extremity, contralateral to the brain lesion (i.e. [16, 17]) or on the less-affected upper extremity, ipsilateral to the brain lesion (i.e. [18, 19]), but not on both hands. The research of bimanual coordination post-stroke may also be lacking because tools that include bimanual functional tasks (such as reviewed above) do not assess bimanual coordination but focus rather on assessing the affected upper extremity. A clinical assessment tool to evaluate bimanual coordination using functional tasks can be valuable for clinicians in stroke rehabilitation.

Therefore, the aim of this paper is to describe the development of a valid and reliable observational tool to assess bimanual coordination; the Bimanual Observation of The Hands (BOTH).

### Development and content validity of BOTH

BOTH is an observation tool to assess bimanual coordination while participants perform bimanual functional tasks. The development and content validity of BOTH follows Kielhofner’s (2006) [20] 10-step process of instrument development, which are described below. Content validity refers to the extent to which a measurement tool accurately captures the domain it is intended to measure [20]. Content validation requires first to conceptually define the domain that is being measured and to specify how it is operationally defined [21]. The content validation of BOTH included reviewing the existing tools that aim to assess a similar construct (as presented above). In addition, we reviewed relevant literature, and we consulted with experts in multiple rounds throughout the instrument’s development [20].

#### Step 1. Identify the need for an instrument

We recognized the need for a tool to assess bimanual coordination of individuals post-stroke, this is described in the Introduction section. The ability to perform bimanual tasks (i.e. tasks that require the use of both hands) has been tested using existing tools, but it does not cover this construct.

#### Step 2. Identify the purpose and the intended population

The purpose of BOTH is to assess bimanual coordination post-stroke. It is suitable to assess the bimanual coordination only of individuals who have some arm-hand capacity (moderate to mild upper extremity motor impairment) of their affected upper extremity post-stroke. The upper extremity subtest of the Fugl-Meyer Motor Assessment (FMA) [22] cutoff score was set to 30/66 points, which is based on the established cutoff scores of the FMA to predict notable, or full upper extremity capacity [23]. To ensure participants also have some finger, hand and wrist movements in order to perform BOTH’s tasks, the FMA ‘Wrist’ and ‘Hand’ sections cutoff score was set to 7/14 points.

#### Step 3: Specify the underlying construct

**Fig 1:**
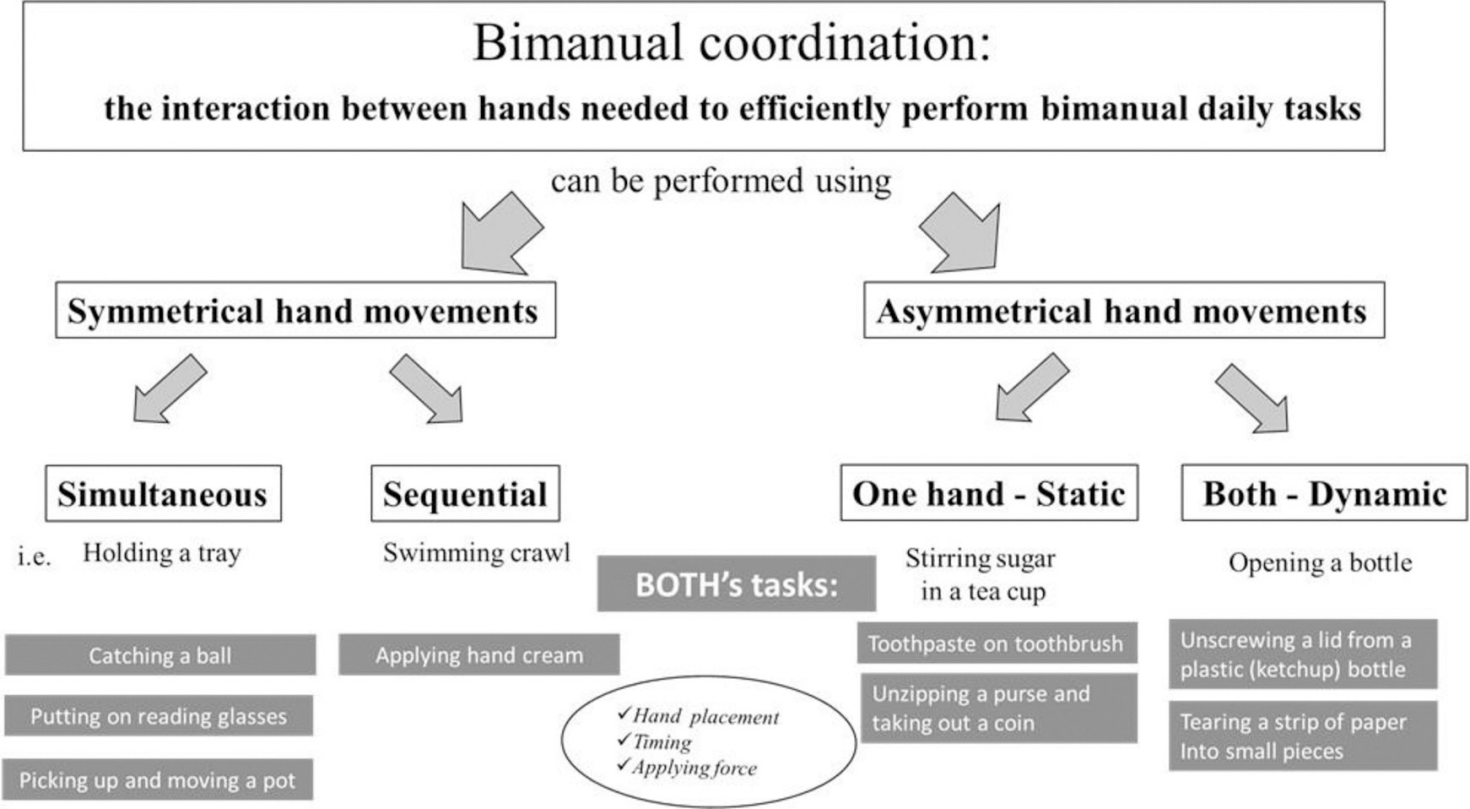
Classification and definition of bimanual coordination that can be performed using symmetrical or symmetrical movements, examples of tasks, BOTH’s tasks and criteria for scoring.

The underlying construct is bimanual coordination; we found two definitions of bimanual coordination. Woytowicz, Whitall, & Westlake (2016) [24] defined two forms of bilateral upper extremity coordination: using symmetric movements [in-phase (i.e., carrying a tray), antiphase (i.e., swing movements while walking) and complex phasing (i.e., drumming)] and asymmetric movements [complementary (i.e., eating with a knife and fork) or independent (i.e., holding a pen while drinking from a cup)]. A similar definition to bimanual actions (not coordination) was provided by Kantak et al. (2017) [15]. According to this definition, bimanual actions include symmetric and asymmetric arm movements, which can be performed by each hand independently (i.e. talking milk and a bowl to the breakfast table) or by performing common goals (i.e. cutting food with a knife and fork). Based on these definitions of bimanual coordination, we defined the following concepts: Bimanual coordination is the interaction between a person’s hands, which is needed to complete bimanual daily tasks efficiently, and which can be performed using symmetrical or asymmetrical hand movements. See Fig 1 to see the differentiation between simultaneous and sequential symmetrical movements and the one-hand-static versus both-dynamic asymmetrical movements and examples. Note that the focus is not on one of the hands or on the role of each hand during the task but on the coordination between the hands.

#### Step 4. Create a plan regarding how the concept will be operationalized & Step 5—Determine how the concept will be operationalized

These steps included identifying the components of bimanual coordination necessary to perform everyday functional tasks with both hands. In order to operationalize the concept of bimanual coordination, we performed a literature review in general and specifically on stroke and summarized the main ideas in the Introduction section. We also aimed to identify the components of bimanual coordination by conducting a task analysis of individuals as they performed functional hand activities, such as cutting wrapping paper with scissors, folding a piece of paper, and inserting it into an envelope. We analyzed videos (from a previous study) of individuals with stroke and healthy individuals performing a complex bimanual task of wrapping a gift and writing a card (See Box 1 for more details). The following criteria for scoring bimanual coordination were defined: Coordination between hands in terms of hand placement, timing of the movement, and the force applied by the hands (See Table 1 for explanations).

**Table 1:** (i) Classification and definition of bimanual coordination that can be performed using either symmetrical or symmetrical movements and examples of tasks, and (ii) Criteria for scoring bimanual coordination.

**Symmetrical hand movements are performed when both hands complete the exact same movement to perform the task, which can be performed simultaneously or sequentially.** Simultaneous symmetrical hand movements are when the hands perform the same movement by applying the same force at exactly the same time. Holding a tray and clapping hands are examples of these movements. Sequential symmetrical hand movements are when the hands perform the exact movements and apply the same force, but the hands follow each other sequentially. Hand movements while swimming, crawling, or by pulling a rope closer to the body, hand after hand, are examples of these movements. **Asymmetrical hand movements are performed to complete a task when each hand plays a different role. These movements can be performed in two ways: one hand is static while the other hand is dynamic during the task or both hands are dynamic.** Asymmetrical movements with one hand static–one hand stabilizes the object (static) while the other hand manipulates the object. Stirring sugar with a spoon while the tea cup is held by the other hand is one example. Asymmetrical dynamic hand movements–when both hands move at the same time (in opposite or the same direction), but each hand performs a different task or holds a different (part of the) object. For example, by opening a bottle (one hand holding the lid and the other hand holding the bottle) and each hand twists in the opposite direction or when cutting a piece of wrapping paper–one hand moves while cutting and at the same time, the other hand moves to stabilize the paper. Some tasks can be performed in both ways. For example, when unscrewing a lid of off a ketchup bottle one hand will hold the lid and one hand will hold the bottle. If performed using asymmetrical dynamic hand movements, each hand twists in the opposite direction to unscrew the lid. If performed using asymmetrical movements with one hand static, the hand on the bottle will be static, stabilizing the bottle, while the other hand will unscrew the lid. The lid will probably be unscrewed faster, when performed using asymmetrical dynamic hand movements, which requires better bimanual coordination. If the bottle is heavy or is placed on a table, perhaps asymmetrical dynamic hand movements are not possible and then asymmetrical movements with one hand static, will be used. Note that our definitions of asymmetrical movements are only of complementary [21] or common goal [15] tasks.
**The criteria for scoring bimanual coordination are as follows:** ***Hand placement*** refers to the location of both hands while holding and manipulating the item. In addition, the placement of the hands in relation to each other is observed. The observers should ask themselves: does the placement of the hands facilitate efficient task performance? As the task progresses, do the hands maintain efficient hand placement in relation to each other? ***Timing of the movement*** refers to the pace of the movement of one hand in relation to the pace of the movement of the other hand. The observers should ask themselves: does the timing promote efficient function? Is one hand faster than the other hand? Is one hand “waiting” for the other hand? ***Applying force*** refers to the force generated by one hand in relation to the force generated by the other hand. The generation of adapted force contributes to efficient bimanual coordination, whereas applying force with one hand that is not adapted to the generation of force from the other hand may negatively impact it. The observers should ask themselves: do both hands grasp the object with adjusted force? Does one hand push harder than the other?

**Box 1. The process of concept operationalized; the task analysis of a bimanual task to identify criteria to rate bimanual coordination**

- In a previous study (unpublished) we administered the Ad-AHA-Stroke [4] to 30 healthy individuals and 42 individuals 6-months post-stroke. As part of the Ad-AHA-Stroke administration, we video-taped these participants wrapping a gift and writing a card. Three authors (DR, NO, SAK) watched these videos and performed task analysis attempting to identify the characteristics of bimanual coordination that can help in defining and rating it.
- We then compiled a list of criteria to rate bimanual coordination. For example, the timing of the initiation of the movement of both hands, the positioning of the palm and fingers, the force used by the hands, the distance of the hands from mid-body, and the location of one hand in relation to the other.
- We sent videos to three experienced OTs who were asked to rate the bimanual coordination of four participants (two healthy and two with stroke) using the list of criteria we compiled.
- Based on their ratings as well as on ours, we narrowed down the list of criteria for bimanual coordination to three: Hand placement, Timing, and Force of the movements.

### Step 6. Decide the format of the instrument

While administering the BOTH, the assessor observes the participant performing functional tasks using bimanual symmetrical and asymmetrical movements. For each task, the assessor rates 0–3 points for the three criteria: Hand placement, Timing, and Applying force.

#### Step 7- Develop the tasks

The bimanual tasks selected for the observation were based on the definitions of bimanual coordination. We selected tasks that are not predominantly performed with the dominant hand and that would be easy and accessible to administer in a clinical setting without the need for sensors or cameras to track movement. These tasks were chosen to represent a range of bilateral movements as defined above and to be quick and efficient to perform. It was also important to select tasks that include available, every-day, and inexpensive equipment. For example, peeling a cucumber, which is a good asymmetrical everyday task was out ruled because it would require keeping fresh cucumbers in the clinic fridge. See the list of current and previous selected tasks in Table 2.

**Table 2:** The tasks included in the different versions of BOTH.

	Version 1	Version 2	Version 3
Used for		Round 1 of the expert validity	Round 2 of the expert validity and for reliability and validity testing
1	Folding a piece of paper and putting in an envelope	Folding a piece of paper and putting in an envelope	-
2	Tearing a strip of paper into small pieces	Tearing a strip of paper into small pieces	Tearing a strip of paper into small pieces
3	Unzipping a pencil case	Unzipping a pencil case	Unzipping a *wallet* and taking out a coin
4	Catching a ball	Catching a ball	Catching a ball
5	Opening a jar of coffee	Opening a jar of coffee	Unscrewing a lid from a plastic (ketchup) bottle
6	Applying hand cream	Applying hand cream	Applying hand cream
7		Rolling a pair of socks	-
8			Applying toothpaste to a toothbrush
9			Putting on reading glasses
10			Picking up and moving a pot

#### Step 8—Develop suitable supporting materials

This process included writing a brief introduction to BOTH, developing administration instructions, defining general scoring criteria as well as specific criteria for each task, creating a scoring sheet, and compiling a list of items needed for the assessment kit to administer BOTH. The manual includes task specific descriptions and scoring on separate sheets. The administration instructions include specific instructions regarding the client’s starting position, set up, items needed, and the wording of the instructions. We also specified what information can be learned from each task and added photos to clearly demonstrate the tasks.

The scoring sheet, which is used during the observation, includes eight tasks; for each task, the three criteria (timing, placement, and force) are scored as 1 = “no”, 2 = “partial”, and 3 = “full” bimanual coordination. The task specific descriptions define what is considered less efficient performance of the task, which can highlight impaired bimanual coordination. For example, if a person needs to adjust their hand placement on the object and try again, or to adjust the force applied or to improve the timing, this indicates performance is less efficient. Also, although task completion success is not directly rated, it helps highlight impaired bimanual coordination. For example, if the eye glasses are placed on the person’s face in a skew manner or if the pot is transferred at an angle, we can learn that bimanual coordination is impaired. The scores of all the tasks are summed up to the total bimanual coordination score (ranging from 24 to 72 points). In addition, a bimanual coordination score for symmetrical tasks (ranging from 12 to 36) and a bimanual coordination score for asymmetrical tasks (ranging from 12 to 36) are calculated. BOTH’s manual and scoring sheet can be downloaded freely from https://english.tau.ac.il/profile/drand.

#### Step 9–10—Pilot the instrument, revise, and develop supporting material

In the early stages of developing the instrument, we selected three symmetrical and three asymmetrical tasks (version 1). After item and supporting material development of BOTH, we approached nine (local and international) expert occupational therapists (OTs) and requested their feedback regarding the *tasks* (do you agree that bimanual coordination is needed to perform these tasks? Should we add or eliminate any tasks?), *criteria for rating* (do you agree that hand placement, timing, and applying force are suitable criteria for bimanual coordination?), *scoring the criteria* (do you agree that the 3-level scoring for each criterion is appropriate?), *screening for suitability to administer BOTH* (how would you define the motor ability of the affected upper extremity?) and *additional feedback*. The OTs provided suggestions and changes were made accordingly. Some tasks were modified (for example, opening a jar of coffee was changed to unscrewing a plastic bottle of ketchup), some tasks were added (for example, putting on eye glasses) and other tasks remained unchanged (for example, catching a ball) (version 2). Table 2 describes the tasks for the different versions of BOTH. We ensured that we have the same number of symmetrical and asymmetrical tasks. Based on feedback received from the OT experts we also clarified the instructions and modified the description and scoring of some of the tasks. All the OTs agreed that the criteria for rating bimanual coordination and scoring of the criteria are correct. Based on their overall feedback, we prepared a revised version of BOTH (version 3), which was used for round 2 of the expert validity and the reliability and validity testing.

## Methods

A cross-sectional study was conducted in two stages: Stage 1: to establish expert validity among OTs to verify that BOTH assesses bimanual coordination. Stage 2: BOTH was administered to individuals with stroke to establish internal reliability (to verify that BOTH’s items covary with each other) and construct validity. Ethics approval was obtained from the University Ethics Committee (#0005185–2, #0006709–2) and the Rehabilitation center’s Helsinki committee (#0108-22-BNZ). First participant recruited October 18^th^ 2022 and last participant recruited March 5^th^ 2024.

### Study population

OTs who did not participate in developing BOTH, with at least three years of clinical experience in stroke rehabilitation were invited to participate. All OTs signed the informed consent form before answering the questions.

Participants with stroke were recruited from an in-patient and an out-patient rehabilitation center and from the community. Participants had moderate to no motor impairment of their affected upper extremity (as verified by a score of at least 30/66 points on the FMA). All participants signed the informed consent form before participation.

### Tools

Stage 1: The ‘Template for assessing content validity through expert judgement’ [25] questionnaire was used to evaluate four different characteristics: sufficiency, clarity, coherence, and relevance, regarding the items (tasks) included in BOTH. This was done separately for the symmetrical items and the asymmetrical items, which were classified into three or four levels [1-does not meet the criterion, 2-low level, 3-moderate level, and 4-high level].

Stage 2: The Purdue pegboard Test [26] was administered to the participants following BOTH. This is a well-known, valid and reliable test of dexterity. The test includes four subtests, we used two of the subtests that requires simultaneous use of both hands. **Both hands placement subtest:** Participants are required to place as many pins as possible down both rows of the pegboard; the number of pairs within 30- seconds is recorded. **Assembly subtest:** Participants are required to use both hands simultaneously while assembling pins, washers and collars; the number of pieces used to assemble within 60 seconds is recorded.

Demographic information was collected from all participants and information regarding the stroke for the participants post stroke. The Functional Independence Measure (FIM) [27] and The Montreal Cognitive Assessment test (MoCA [28] were used to characterize the sample of individuals post-stroke in terms of independence in daily living and cognition (respectively).

### Data analysis

Descriptive statistics were used to describe the sample. The incidence and the percentage of the ratings of the item characteristics were calculated. Cronbach’s alpha (95% Confidence Interval (CI)) was used to check the internal consistency of BOTH. Alpha values that approach .90 are indications of high homogeneity of a scale [20]. The item-total correlation was verified for BOTH total score and sub-scores [20] using Spearman correlations. Spearman correlations were also used to assess correlations between BOTH (total, symmetrical, asymmetrical) scores and the two subtests of the Purdue.

## Results

### Stage 1: Expert validity

Eight OTs [mean (SD) age—38.2 (6), with 11.8 (7.2) years of experience] rated the four characteristics of BOTH version 2. After improving the items based on their feedback, we asked another three OTs [mean (SD) age—30.6 (4.5), with 4.5 (1.8) years of experience] to rate it as well. As shown in Table 3, the ratings of the four characteristics improved. The sufficiency characteristic of the symmetrical items was further improved by consulting with another expert OT.

**Table 3:** Expert validity was performed in two rounds by 11 OTs.

	Round 1 Version 2 (n = 8)				Round 2 Version 3 (n = 3)			
Symmetrical items								
	**1**	**2**	**3**	**4**	**1**	**2**	**3**	**4**
	N (%)	N (%)	N (%)	N (%)	N (%)	N (%)	N (%)	N (%)
Sufficiency	12.5	0	12.5	75	0	33.3	0	66.7
Clarity	0	12.5	87.5		0	0	100	
Coherence	0	25	0	75	0	0	0	100
Relevance	0	25	12.5	62.5	0	33.3	33.3	33.3
Asymmetrical items								
Sufficiency	0	0	12.5	87.5	0	0	0	100
Clarity	0	37.5	62.5		0	0	100	
Coherence	0	12.5	12.5	75	0	0	0	100
Relevance	0	25	12.5	62.5	0	0	33.3	66.7

1-does not meet the criterion, 2-low level, 3-moderate level, and 4-high level.

Clarity is rated 1–3 but other items are rated 1–4. A higher score indicates that the characteristics are better.

#### Stage 2: Fifty-one individuals with stroke (16 women) completed the BOTH

Thirty-six participants were in subacute rehabilitation, the rest lived at home. Participants were aged 33 to 88 years (mean (SD) 61.9 (10.7). They ranged in their independence in daily living (FIM ranged from 60–125 points, mean (SD)– 103.7 (16.3) and in their cognitive status (MoCA 16–30, mean (SD) 23.9 (3.3)/30 points). Of the 51 participants, 30 also underwent the Purdue pegboard test.

### Internal reliability

BOTH showed good internal consistency. Cronbach’s alpha value (95% CI) for the eight items of BOTH was α = .949 (.924-.968), α = .923 (.881-.952) for the asymmetrical items and .897 (.842-.937) for the symmetrical items. In addition, the item-total correlation was verified for BOTH’s total score and sub-scores (see Table 4). Significant moderate-high to high correlations were found between the eight tasks to BOTH’s total score (r = .865-.956, p < .001), the four symmetrical tasks to the symmetrical score (r = .853-.906, p < .001) and the four asymmetrical tasks to the asymmetrical score (r = .889-.917, p<001).

**Table 4:** Item-total correlations and item-symmetrical and asymmetrical scores of BOTH (n = 51).

Tasks that require		Total score	Symmetrical score	Asymmetrical score
Symmetrical movements	Putting on reading glasses	.826**	.853**	-
	Moving a pot	.862**	.861**	-
	Catching a ball	.857**	.906**	-
	Applying hand cream	.828**	.885**	-
Asymmetrical movements	Tearing a strip of paper	.868**	-	.906**
	Applying toothpaste	.956**	-	.894**
	Unzipping a wallet	.904**	-	.917**
	Unscrewing a lid off a bottle	.865**	-	.889**
	Total score		.966**	.970**

### Construct validity

BOTH’s total and sub-scores were strongly significantly correlated with the Purdue subtests. See Table 5

**Table 5:** Construct validity (N = 30).

	BOTH		
Purdue Pegboard Test	Total score	Symmetrical score	Asymmetrical score
**Both hands placement subtest**	.840**	.771**	.832**
**Assembly subtest**	.801**	.765**	.800**

## Discussion

This study described the need and the development process of a new observational tool to assess bimanual coordination of individuals post-stroke using functional tasks. Expert validity helped improve the tool and modified the tasks. BOTH was then administered to individuals post-stroke at different stages. Excellent internal reliability and construct validity were found.

Bimanual coordination is a crucial component of efficient bimanual functioning. Despite this fact it has not been the focus of research post-stroke, nor has improving bimanual coordination been the focus of upper extremity therapy post-stroke. Stroke research has mainly focused on the more affected upper extremity, aiming to better understand the characteristics that can be addressed to improve daily hand function post-stroke. Existing tools assess the ability of the affected upper extremity to perform unilateral and bilateral tasks, bimanual activity, bimanual performance, or bimanual function [8, 11, 12], however bimanual coordination is not assessed.

The current version of BOTH is definitely a combined effort to formulate an observational tool that can be used with patients undergoing rehabilitation as well as for future research. The multiphase development process was challenging, especially due to the lack of agreed-upon conceptual definitions of bimanual coordination and what it entails. Establishing a clear conceptual definition is essential in the process of tool development [21]. We defined bimanual coordination as the interaction between a person’s hands, needed to complete bimanual daily tasks efficiently. This can be performed using (simultaneous or sequential) symmetrical or asymmetrical hand movements. Asymmetrical hand movements may include one hand which is static or both hands, which are dynamic. Our definitions are based on previous definitions of common goal movements [15] as well as symmetrical and asymmetrical complementary movements [24] but the differentiation of static versus dynamic asymmetrical movements is new.

Expert validity is one aspect of content validation to confirm that the tool is coherent and that it represents what is supposed to be assessed [20]. We asked OTs to rate four different characteristics: sufficiency, clarity, coherence, and the relevance of the symmetrical and asymmetrical tasks. Based on their feedback, we modified the tasks of BOTH, helping to obtain better agreement. Relevance of the tasks did not achieve 100% agreement because some OTs felt that the task ‘Tearing a strip of paper into small pieces’ was not a relevant everyday task. Despite this feedback, we decided to include the task because it provides information regarding the interplay between the hands (similar to using scissors to cut wrapping paper, but easier) that is not picked-up by the other tasks.

The excellent internal reliability supports BOTH as a tool that includes items that assess the same construct; bimanual coordination. Four tasks require symmetrical movements and four tasks involve asymmetrical movements. Within these, tasks entailed different types of movements. For example, for the symmetrical tasks, catching a ball requires fast movement, putting on reading glasses require delicate movements and for moving a pot, gross movements and strength are required. Interestingly, despite the fact that different types of movements are used, all four symmetrical tasks are strongly correlated to the symmetrical sub-score. For the asymmetrical tasks, the one-hand-static and both dynamic tasks were strongly correlated to the asymmetrical and total scores.

When developing a tool, obtaining feedback from colleagues and patients is important. As part of the development of BAM [8], for example, focus groups of OTs and patients were carried out. We did not do this, instead we obtained ongoing feedback from OTs who have clinical experience in stroke rehabilitation. Group discussions of OTs could have been very beneficial as well as of patients. Patient’s feedback should be collected in the future when using the BOTH for research.

Construct validity of BOTH was supported by correlating bimanual coordination as assessed using BOTH to two Purdue Pegboard subtests. Purdue Pegboard Test, is not defined as a tool to assess bimanual coordination but two of the four subtests do include the use of both hands simultaneously. These timed tasks include picking up and placing tiny pins to assess dexterity. Participants who inserted more pairs of pins and used more pieces for assembling, were observed by the OTs to have better bimanual coordination using BOTH. Our participants had moderate to good motor ability and therefore could perform the Purdue pegboard test but many participants post-stroke are not able to perform because of the small pins, which require good finger movements. BOTH tasks are functional, familiar and easier to perform than the Purdue. Further research with other tools is needed to validate BOTH.

Impaired bimanual coordination, assessed by BOTH, may help explain the discrepancy often seen between (higher) capacity and (limited) actual daily use of this hand [29, 30] during daily living, which entails mainly bimanual tasks. The measurement of bimanual coordination in a clinical setting may encourage clinicians to address bimanual coordination in their therapeutic sessions. Perhaps this will lead to developing novel interventions to improve bimanual coordination and functioning post-stroke. Having a reliable and valid tool to assess bimanual coordination might also encourage research on this important component, which has been previously neglected.

We acknowledge certain limitations. The development of BOTH did not include interviews or focus groups with individuals with stroke. This might have led to insights regarding bimanual coordination and the perceived impact of bimanual coordination on daily functioning. In addition, a larger sample of individuals post stroke are needed to further establish the validity of BOTH as a tool to assess bimanual coordination. The inter-rater and test-re-test reliability need to be established as well, before BOTH can be used for clinical and research purposes.

## Conclusions

BOTH was developed to address the lack of clinical and practical tools to assess bimanual coordination post-stroke using functional tasks. The expert validity of BOTH was established and the preliminary internal reliability and construct validity were demonstrated. In the future, BOTH can be used for both clinical and research purposes to address impaired bimanual coordination post-stroke.

## Supporting information

S1 Data (SAV)
